# Platelet Adhesion and Thrombus Formation in Microchannels: The Effect of Assay-Dependent Variables

**DOI:** 10.3390/ijms21030750

**Published:** 2020-01-23

**Authors:** Mariangela Scavone, Silvia Bozzi, Tatiana Mencarini, Gianmarco Podda, Marco Cattaneo, Alberto Redaelli

**Affiliations:** 1 Unità di Medicina 2, ASST Santi Paolo e Carlo, Dipartimento di Scienze della Salute, Università degli Studi di Milano, 20142 Milan, Italy; mariangela.scavone@guest.unimi.it (M.S.); gianmarco.podda@unimi.it (G.P.); marco.cattaneo@unimi.it (M.C.); 2 Department of Electronics, Information and Bioengineering, Politecnico di Milano, 20133 Milan, Italy; silvia.bozzi@polimi.it (S.B.); tatiana.mencarini@mail.polimi.it (T.M.)

**Keywords:** platelets, thrombus formation, microfluidics, flow assays, platelet adhesion, platelet aggregation, shear rate, antiplatelet agents

## Abstract

Microfluidic flow chambers (MFCs) allow the study of platelet adhesion and thrombus formation under flow, which may be influenced by several variables. We developed a new MFC, with which we tested the effects of different variables on the results of platelet deposition and thrombus formation on a collagen-coated surface. Methods: Whole blood was perfused in the MFC over collagen Type I for 4 min at different wall shear rates (WSR) and different concentrations of collagen-coating solutions, keeping blood samples at room temperature or 37 °C before starting the experiments. In addition, we tested the effects of the antiplatelet agent acetylsalicylic acid (ASA) (antagonist of cyclooxygenase-1, 100 µM) and cangrelor (antagonist of P2Y_12_, 1 µM). Results: Platelet deposition on collagen (I) was not affected by the storage temperature of the blood before perfusion (room temperature vs. 37 °C); (II) was dependent on a shear rate in the range between 300/s and 1700/s; and (III) was influenced by the collagen concentration used to coat the microchannels up to a value of 10 µg/mL. ASA and cangrelor did not cause statistically significant inhibition of platelet accumulation, except for ASA at low collagen concentrations. Conclusions: Platelet deposition on collagen-coated surfaces is a shear-dependent process, not influenced by the collagen concentration beyond a value of 10 µg/mL. However, the inhibitory effect of antiplatelet drugs is better observed using low concentrations of collagen.

## 1. Introduction

Platelets play a central pathogenic role in thrombosis; they aggregate at sites of atherosclerotic plaques, forming thrombi that can occlude the lumen of the artery [1,2,3]. In coronary arteries, this process causes acute coronary syndromes, which are a major cause of morbidity and mortality [4]. Atherosclerotic plaques favor the formation of thrombi by exposing thrombogenic surfaces, to which platelets adhere, and by causing stenosis of the arterial lumen, which contributes to platelet activation by increasing the shear rate of blood flow [5,6]. Patients with acute coronary syndrome are treated with antiplatelet drugs, inhibiting cyclooxygenase-1 (COX-1) (acetylsalicylic acid) and P2Y_12_ receptors (clopidogrel, ticagrelor or prasugrel).

Microfluidic flow chambers (MFCs) designed to observe platelet adhesion and thrombus formation on surfaces coated with adhesive proteins, such as collagen, have become a valuable tool for research in hemostasis and thrombosis [7,8,9]. These devices can characterize platelet function under flow with low blood volume requirements and controlled conditions. They can mimic the anatomy of healthy and stenotic blood vessels [7,10], recreate a range of physiological and pathological shear stress conditions [11,12,13] and investigate platelet accumulation over different adhesive proteins [14,15]. Furthermore, these devices allow to study the inhibitory effect of antiplatelets agents on platelet adhesion and thrombus formation [16,17].

However, as a consequence of their high flexibility, MFCs are poorly standardized [18]. Limited studies have been performed to assess the variability of platelet accumulation in relation with the experimental variables. With regards to platelet deposition on collagen, most of the works have focused on the role of the mechanical stresses, reporting on the significant shear-rate dependency of the platelet adhesion and thrombus formation processes, e.g., [12,19,20]. The sensitivity to collagen concentration was investigated by Savage et al. [21] and Neeves and colleagues, who performed the most comprehensive study on the sources of variability in platelet accumulation on collagen, exploring also the effect of phenotypic and genetic factors [19]. The inhibitory effect of antiplatelet drugs on platelet accumulation has been studied by Diamond’s research group in a number of works [16,17,22]. Despite these studies and some attempts to define common protocols [18,23], no real standardization has been achieved and the effect of a number of assay-dependent variables still remains to be fully elucidated. 

Given this background, the current study aims at investigating a number of critical aspects in the study of platelet adhesion and thrombus formation with MFCs: The effect of the storage temperature of the blood samples before testing; the influences of wall shear rate and of the concentration of the adhesive protein collagen in the surface coating solution; and the inhibitory effect of some antiplatelet drugs.

## 2. Results

### 2.1. Effect of Blood Storage Temperature 

**Figure 1:**
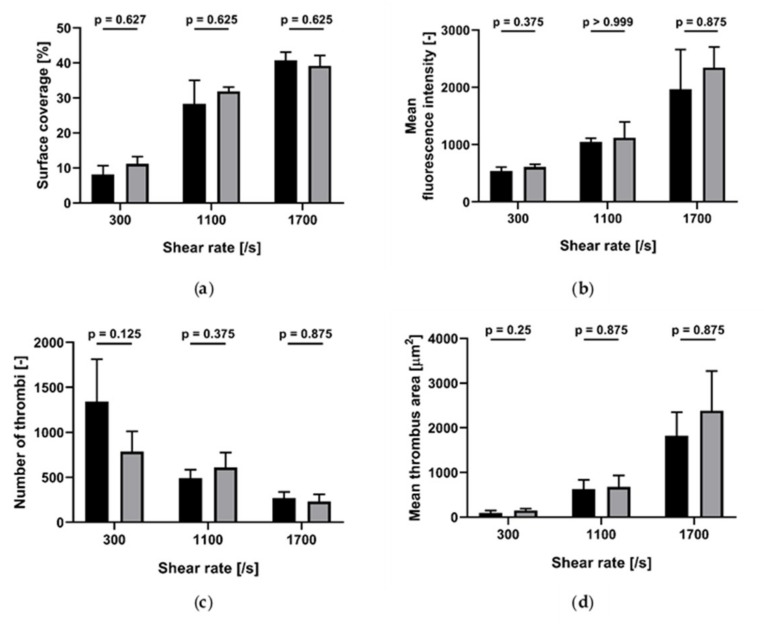
Effect of storage temperature (room temperature (RT) and 37 °C) on platelet accumulation on collagen (200 µg/mL)-coated channels at different wall shear rates (*n* = 4). Black: RT; grey: 37 °C. (**a**) Surface coverage; (**b**) mean fluorescence intensity; (**c**) number of thrombi; (**d**) mean thrombus area. Data are represented as column bar graphs, with means ± standard errors of the mean and analyzed by Wilcoxon matched pairs.

We measured the effect of blood storage temperature at room temperature (RT) and 37 °C on platelet accumulation on collagen (200 µg/mL)-coated channels, at 300/s, 1100/s and 1700/s wall shear rates (Figure 1). In all conditions tested, no statistical difference was found between the results obtained at RT and 37 °C.

### 2.2. Effect of Wall Shear Rate

**Figure 2:**
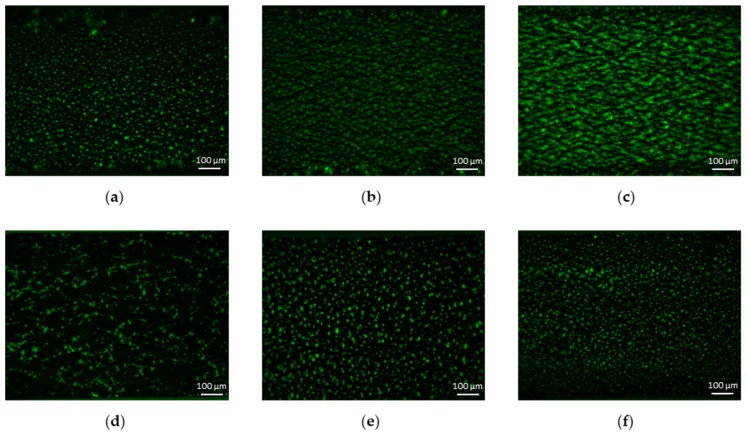
Representative images (6.3×) of platelet accumulation at different shear rates and collagen concentrations. Images are acquired after 4 min of perfusion. Flow is from left to right. The first row shows platelet accumulation over collagen (200 µg/mL)-coated perfusion chamber at (**a**) 300/s, (**b**) 1100/s and (**c**) 1700/s. The second row shows platelet accumulation at 300/s for collagen concentrations equal to (**d**) 10 µg/mL, (**e**) 50 µg/mL and (**f**) 100 µg/mL.

**Figure 3:**
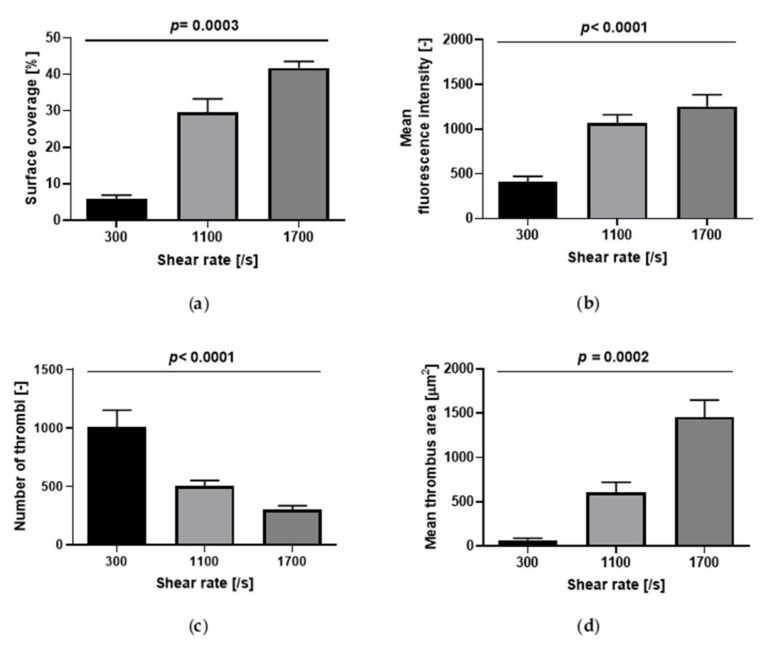
Effect of wall shear rate on platelet accumulation on collagen (200 µg/mL)-coated microchannels (*n* = 7). (**a**) Surface coverage; (**b**) mean fluorescence intensity; (**c**) number of thrombi; (**d**) mean thrombus area. Data are represented as column bar graphs, with means ± standard errors of the mean, and analyzed by Kruskal–Wallis tests or ANOVA tests as appropriate. Internal Contrasts: (**a**) 300/s vs. 1700/s, *p* < 0.001; (**b**) 300/s vs. 1100/s *p* < 0.001; 300/s vs. 1700/s, p < 0.001; (**c**) 300/s vs. 1100/s *p* < 0.01; 300/s vs. 1700/s, *p* < 0.001; (**d**) 300/s vs. 1700/s, *p* < 0.001.

Platelet accumulation, as measured by surface coverage and fluorescence intensity, increased as a function of wall shear rate (Figure 2; Figure 3a,b). The number of thrombi significantly decreased while the mean thrombus area significantly increased as a function of wall shear rate (Figure 2 and Figure 3c,d).

### 2.3. Effect of Collagen Concentration

**Figure 4:**
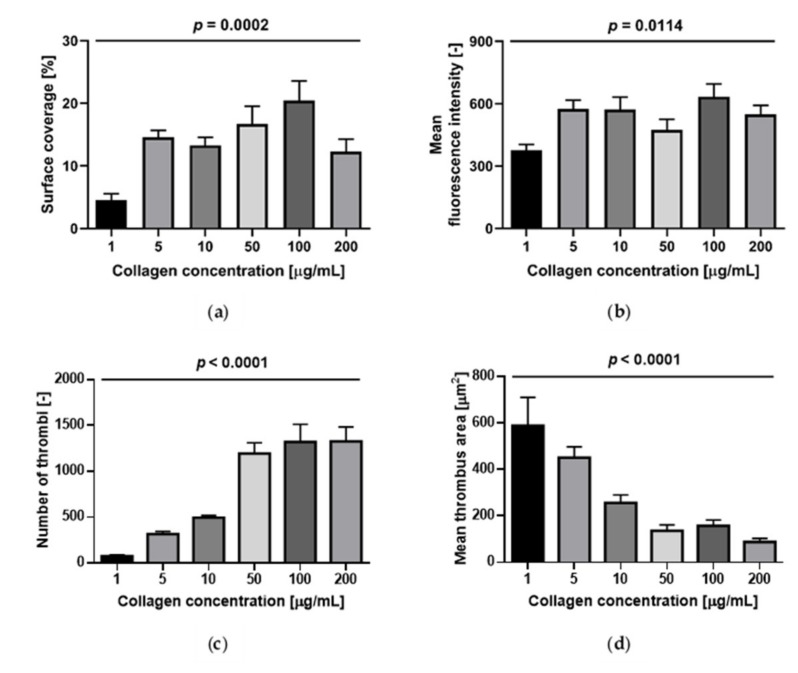
Effect of collagen concentration on platelet accumulation at 300/s (*n* = 7). (**a**) Surface coverage; (**b**) mean fluorescence intensity; (**c**) number of thrombi; (**d**) mean thrombus area. Data are represented as column bar graphs, with means ± standard errors of the mean, and analyzed by ANOVA tests plus Bonferroni’s multiple comparison post-hoc tests. Internal Contrast: (**a**) 1 vs. 5 µg/mL of collagen, *p* < 0.05; 1 vs. 50 µg/mL of collagen, *p* < 0.01; 1 vs. 100 µg/mL of collagen, *p* < 0.001; (**b**) 1 vs. 100 µg/mL of collagen, *p* < 0.05; (**c**) 1 vs. 50, 100 and 200 µg/mL of collagen, *p* < 0.001; 5 vs. 50, 100 and 200 µg/mL, *p* < 0.001; 10 vs. 50 µg/mL of collagen, *p* < 0.01; 10 vs. 100 and 200 µg/mL of collagen, *p* < 0.001; (**d**) 1 vs. 10 µg/mL of collagen, *p* < 0.01; 1 vs. 50,100, 200 µg/mL, *p* < 0.001; 5 vs. 50, 200, *p* < 0.01; 5 vs. 100 µg/mL, *p* < 0.05.

Surface coverage and fluorescence intensity at a shear rate of 300/s were affected by collagen concentration: They were lowest at 1 µg/mL and slowly increased with increasing collagen concentration up to 100 µg/mL; at 200 µg/mL the surface coverage tended to decrease (Figure 4). The internal contrast showed that there were no statistically significant differences over the range 5 to 200 µg/mL (Figure 2 and Figure 4a,b). The number of thrombi significantly increased while their area significantly decreased as a function of collagen concentrations from 1 to 200 µg/mL (Figure 2 and Figure 4c,d).

### 2.4. Effect of ASA on Platelet Accumulation

**Figure 5:**
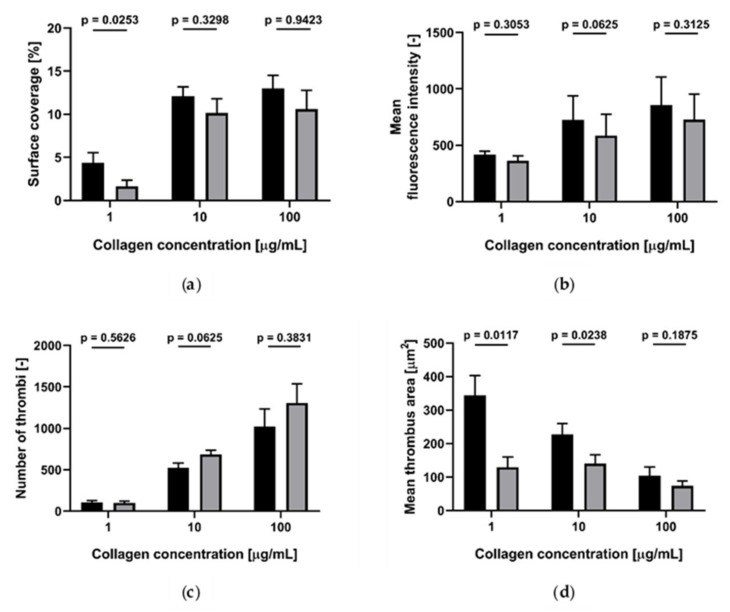
ASA inhibition of platelet accumulation at 300/s for different collagen concentrations (*n* = 5). Black: without ASA; grey: with ASA added in vitro. (**a**) Surface coverage; (**b**) mean fluorescence intensity: (**c**) number of thrombi; (**d**) mean thrombus area. Data are represented as column bar graphs, with means ± standard errors of the mean, and analyzed by Wilcoxon tests or t-tests as appropriate.

- Collagen Concentration = 1 µg/mL. ASA (100 µM) induced a statistically significant decrease in surface coverage (4.4% vs. 1.6%; *p* = 0.025) and mean thrombus area, but did not significantly change mean fluorescence and number of thrombi (Figure 5).
- Collagen Concentration = 10 µg/mL. ASA (100 µM) did not induce a statistically significant change in surface coverage, tended to reduce mean fluorescence intensity and to increase the number of thrombi, but differences were borderline statistically significant and significantly decreased the mean thrombus area (Figure 5).
- Collagen Concentration = 100 µg/mL. ASA (100 µM) did not induce statistically significant modifications of surface coverage, fluorescence intensity, number of thrombi or mean thrombus area (Figure 5).

### 2.5. Cangrelor Inhibition of Platelet Accumulation

**Figure 6:**
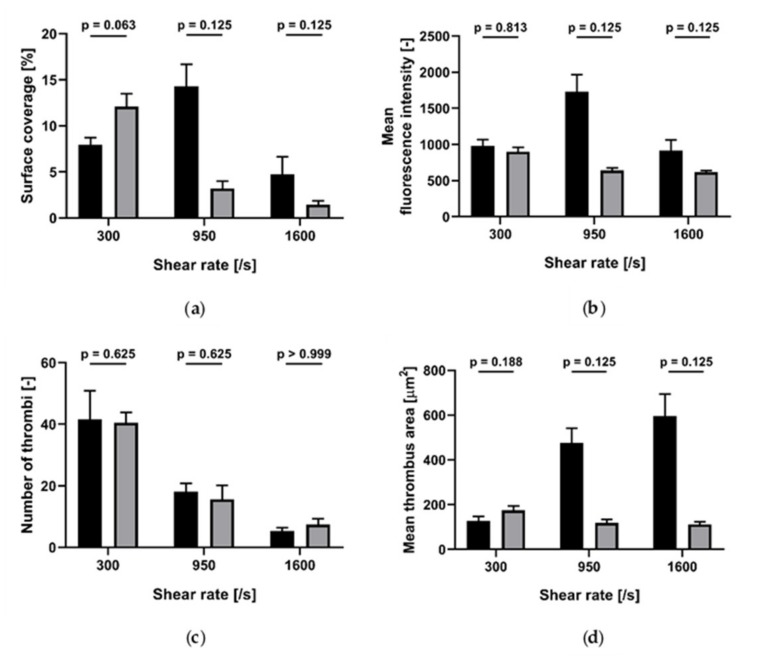
Effect of cangrelor at different shear rates on platelet accumulation on collagen (10 µg/mL)-coated perfusion chamber (*n* = 5). Black: without cangrelor; grey: with cangrelor added in vitro. (**a**) Surface coverage; (**b**) mean fluorescence intensity; (**c**) number of thrombi; (**d**) mean thrombus area. Data are represented as column bar graphs, with means ± standard errors of the mean, and analyzed by Wilcoxon matched pairs.

**Figure 7:**
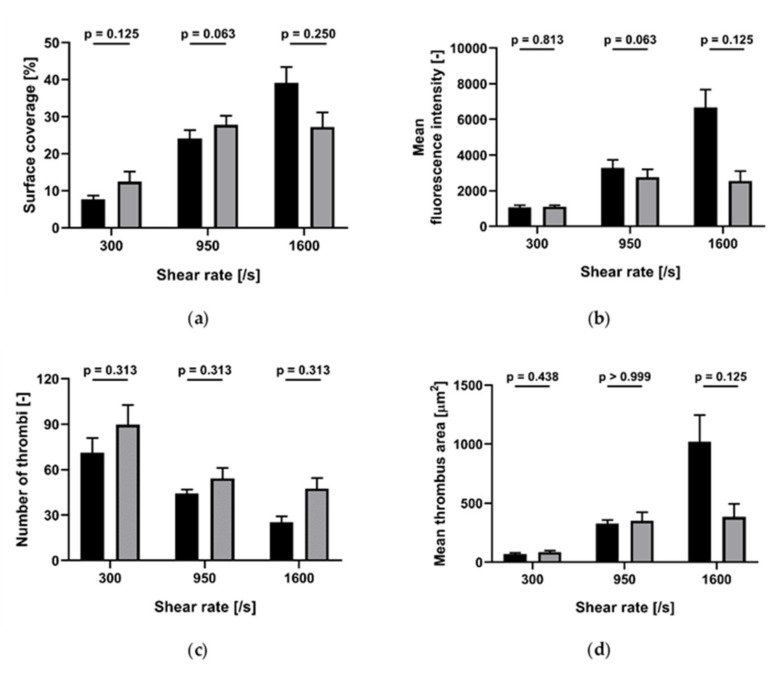
Effect of cangrelor at different shear rates on platelet accumulation on collagen (100 µg/mL)-coated m (*n* = 5). Black: without cangrelor; grey: with cangrelor added in vitro. (**a**) Surface coverage; (**b**) mean fluorescence intensity; (**c**) number of thrombi; (**d**) mean thrombus area. Data are represented as column bar graphs, with means ± standard errors of the mean, and analyzed by Wilcoxon matched pairs.

Collagen concentrations of 10 and 100 µg/mL. At shear rate of both 300/s and 1600/s, cangrelor (1 µM) did not induce a statistically significant reduction of surface coverage, mean fluorescence intensity, number of thrombi or mean thrombus area (Figure 6 and Figure 7).

## 3. Discussion

Our study aimed at investigating different critical aspects of microfluidic platelet adhesion assays on collagen type I: (I) the storage temperature of blood before perfusion; (II) the wall shear rate; and (III) the concentration of collagen in the buffered solution used for coating. In addition, our study aimed at assessing the effect of antiplatelet agents targeting COX-1 (acetylsalicylic acid) and P2Y_12_ (cangrelor) on platelet accumulation under the different experimental conditions considered in our study.

To this purpose, we developed a microfluidic device following the design recommendations given by [24], performed a number of experiments following the protocols suggested by [18] and evaluated the results by comprehensive image analysis, which included not only the typical outputs, such as surface coverage and mean fluorescence intensity, but also the number and area of thrombi, which, as outlined by [25], may change as a function of local flow conditions and platelet function.

Although it has been recommended to store blood samples for platelet function assays at room temperature [26], in our experiments no difference between the storage temperature of blood (at room temperature versus 37 °C) was found for all the WSR studied.

Platelet accumulation was a strongly shear-dependent process, as already observed by many authors, e.g., [19,25]. In our experiments, at 300/s several small and circular thrombi formed, whereas at higher shear rates (1100 and 1700/s), platelet aggregates elongated twofold in the direction of the flow. The same shear dependency of the morphology of platelet aggregates was found by Colace et al. [25], who calculated the width and length of growing thrombi on collagen in the range of shear rates from 100/s to 2000/s. Platelet accumulation increased with increasing shear rate, not only in the horizontal plane (i.e., the glass plane) as measured by surface coverage, but also vertically in the third dimension, as indicated by mean fluorescence intensity. All previous studies reported the same result despite each work observing the peak platelet deposition at a different shear rate: 300/s [19,20], 500/s [12] and 1500/s [21].

Collagen surface density influenced thrombus formation up to a threshold equal to 1 µg/mL with regard to the amount of platelet deposition (measured by SC and FI) and to 5 µg/mL for the variables quantifying the aggregate morphology (number of thrombi and mean thrombus area). At lower concentrations of collagen (1 and 5 µg/mL), a few dispersed, large thrombi have formed, while at higher concentrations (> 10 µg/mL) many small aggregates are present. Similar results were found by Savage et al. [21] who observed an increase in the total volume of thrombi from 0.01 µg/mL to 0.1 µg/mL and no further changes beyond 0.1 µg/mL, as well as by Neeves et al. [19] who observed a significant difference in surface coverage only up to a collagen concentration of 50 µg/mL.

Under our experimental conditions (shear rate equal to 300/s), ASA reduced surface coverage only on surfaces coated with the lowest concentration of collagen (1 µg/mL) but significantly decreased the mean thrombus area both at 1 µg/mL and 10 µg/mL. This is in agreement with Li and Diamond [22] who found that ex-vivo addition of ASA results in smaller platelet aggregates compared to responses measured with no drugs. In the same work, the authors also reported a decrease in total platelet accumulation (measured in terms of fluorescence intensity) over collagen at 200/s following high dose (500 µM) ASA addition, but the collagen concentration was not provided. The effect of ASA is known to be relevant only after some minutes of perfusion [16,17], when secondary aggregation mediated by thromboxane occurs, while initial platelet adhesion to collagen is not influenced by the presence of aspirin. This suggests that the perfusion time of our experiments should probably have been longer to observe the ASA inhibitory effect at high collagen concentrations. Cangrelor, an antagonist of the P2Y_12_ receptor, did not cause any significant inhibition of platelet accumulation at any shear rate and concentration of collagen. However, a distinct trend was observed at the higher shear rates (950/s and 1600/s), indicating a marked decrease in surface coverage, fluorescence intensity and mean thrombus area due to the presence of cangrelor. This suggests that a statistical significance would probably be reached by increasing the number of experiments.

In summary, platelet deposition on collagen type I (I) is not affected by the storage temperature of the blood before perfusion (room temperature vs. 37 °C); (II) was a shear-dependent process in the range between 300/s and 1700/s; and (III) is influenced by the collagen concentration used to coat the microchannels up to a concentration of 5 µg/mL. Antiplatelet agents did not show statistically significant inhibitory effects on platelet interaction with collagen-coated surfaces, except for ASA at low concentrations of collagen.

By carefully examining the effect of several assay-dependent variables on platelet deposition on surfaces coated with collagen type I in a microfluidic device, we believe to have characterized different aspects of thrombus formation that should be taken into account before approaching these experiments. 

## 4. Materials and Methods

### 4.1. Enrollment of Healthy Control Subjects

Healthy subjects (*n* = 15, 3 males; age range: 19–39 years) were recruited among workers of ASST Santi Paolo e Carlo and Università degli Studi di Milano. All studied subjects abstained from drugs known to affect platelet function for at least 10 days before enrolment. The study was approved by the institutional ethical committee of ASST Santi Paolo e Carlo; all subjects signed their informed consent.

### 4.2. Blood Sampling

Blood samples were collected in the morning from an antecubital vein using a 21-gauge butterfly needle with minimal stasis. The first 3 mL of blood was collected into K-EDTA tubes (Becton Dickinson vacutainer, North Ryde, NSW, Australia) for a complete blood count; the following 10–15 mL was collected in 250 µg/mL INN-desirudin (Canyon Pharmaceuticals, London, United Kingdom), gently mixed, and allowed “to rest” at room temperature for 15 min before use.

### 4.3. Fabrication of Microfluidic Devices

**Figure 8:**
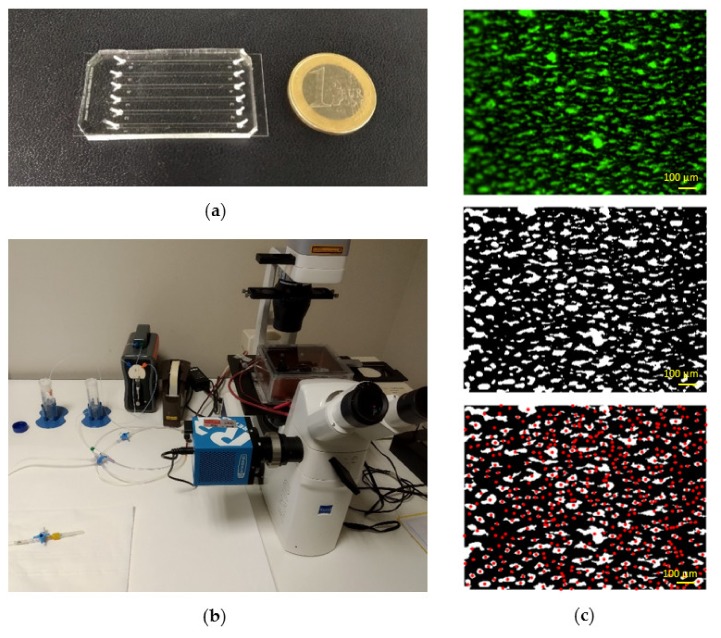
Photograph of the microfluidic chip (**a**) and of the experimental set-up (**b**). Results of the image-processing algorithm (**c**), from top to bottom: original image (6.3×), segmented binary image and identification of platelet aggregates (red dots represent individual thrombi).

Microfluidic devices (Figure 8a) consisted of six independent channels (1000 μm wide, 100 μm high and 3.2 cm long). Channels were designed according to previous recommendations, which suggested an aspect ratio of 10:1 (width:height) in order to reduce wall effects and obtain homogenous platelet distribution across the width of the channel [12,19]. Microfluidic devices were fabricated in polydimethylsiloxane (PDMS, Sylgard™ 184, Dow Corning, Midland, MI, USA) from silicon masters using standard soft lithography techniques. PDMS was prepared by mixing pre-polymer and curing agent in a ratio 10:1 (w:w), degassed, poured over the master mold and cured at 80 °C for 3 h. Inlet and outlet fluidic ports were punched with a 1.5 mm diameter biopsy puncher. PDMS chips were permanently bonded to #0.6 microscope cover glasses via air plasma treatment.

### 4.4. Perfusion Experiments

Each microfluidic channel was incubated with Horm fibrillar collagen type I (Mascia Brunelli, Milano, Italy) at 4 °C overnight. An isotonic glucose solution (pH 2.7–2.9) was used to achieve the desired collagen concentration (1, 5, 10, 50, 100 or 200 µg/mL). Collagen pattering was obtained by pipetting 2.2 μL into the outlet port of the flow chamber. This allowed to fill only about 2/3 of the channel, leaving uncoated the first one-third. In this way, platelets were prevented from adhering in the proximity of the channel entry, where flow disturbances can induce altered platelet accumulation affecting downstream thrombus formation. Then, it was rinsed with filtered phosphate buffer saline (PBS). Before perfusion, it was incubated with 1% bovine serum albumin in PBS for 30 min at room temperature, to passivate the chamber surface that had not been coated by collagen. Subsequently, each channel was washed with PBS, connected with the flow system and checked for the absence of air bubbles. The microfluidic device was then placed on the stage of an inverted fluorescence microscope (Axiovert A1 FL, Zeiss, Milan, Italy) equipped with a 16-bit camera (Sony ICX-674 CCD Camera, Crisel Instruments, Rome, Italy) (Figure 8b). Fluorescence microscopy was performed with a mercury light source (HBO 50 AC L1, Zeiss, Milan, Italy) and a FITC filter set.

Blood samples were incubated with the green-fluorescent lipophilic dye 3,3’ Dihexyloxacarbocyanine Iodide (DiOC6, 1 µM; ThermoFisher Scientific, Milan, Italy), which tags platelets, at room temperature for 15 min. In most experiments, blood samples were kept at room temperature before testing; in some experiments, samples were divided in two equal parts, of which one was kept at RT and one at 37 °C for a maximum of 4h. In some experiments, 100 µM lysine-ASA (Flectadol, Sanofi-Aventis, Milan, Italy) or 1 µM cangrelor (The Medicines Company, Parsippany-Troy Hills, NJ, USA) was added to blood samples at RT for 10 or 3 min, respectively. Before the beginning of the experiments, whole blood samples that had been kept at RT were incubated at 37 °C for 3 min and then perfused for 4 min using a programmable syringe pump (Mirus Evo Nanopump, Cellix, Crisel Instruments, Rome, Italy) through Tygon tubes (0.5 mm inner diameter) inserted into the fluidic ports (Figure 8b). All experiments were performed at 37 °C within three hours from blood collection.

To investigate the effect of collagen surface density on platelet adhesion and thrombus formation, blood was perfused at 300/s over surfaces coated with buffered solutions containing 6 different collagen concentrations: 1, 5, 10, 50, 100 and 200 μg/mL (*n* = 7). To assess the effect of wall shear rate, blood was perfused over collagen (200 μg/mL) at three flow rates, corresponding to 300/s, 1100/s and 1700/s wall shear rates (*n* = 7). Wall shear rate *γ* was related to volumetric flow rate *Q* through the following equation:

(1) $$Q=\;\frac{\gamma wh^{2}}{6}$$

where *w* and *h* refer to the width and height of the microfluidic channel, respectively.

Finally, the inhibitory effect of two antiplatelet drugs (ASA and cangrelor) was assessed under different flow conditions and collagen concentrations. ASA inhibition of platelet accumulation was investigated at 300/s and collagen concentrations equal to 1, 10 and 100 μg/mL (*n* = 5). The effect of cangrelor was evaluated at 300/s, 950/s and 1600/s with collagen concentrations of 10 μg/mL and 100 μg/mL.

### 4.5. Image Acquisition and Analysis

At the end of blood perfusion (4 min) an image was captured in the zone immediately after the beginning of the collagen patch. Image acquisition was performed using a 10× objective lens and a 0.64x video adapter, through the MicroManager software. The field view allowed to capture the full width of the channel. Real-time platelet accumulation was also monitored by acquiring an image every 2 s over the duration of the experiment.

Surface coverage, mean fluorescence intensity, number of thrombi and mean thrombus area were calculated using a custom MATLAB (MathWorks, Natick, MA, USA) script. To limit wall effects, images were cropped down to 80% of the channel width, where the wall shear rate is nearly uniform, within 5% of the value at the center [24]. Mean fluorescence intensity was estimated by averaging all the intensity values of the cropped image. Grayscale images were then converted into binary images by an automatic image thresholding method in order to eliminate operator bias. The Otsu’s algorithm [27] was chosen as it provided a better performance than other global thresholding techniques (e.g., the triangle method). Otsu’s algorithm is a statistical thresholding method, which partition the image intensity histogram into classes so that the between-class variance is maximized. After the thresholding operation, all the segmented images were checked one by one and when inconsistent results were found a background correction of the raw image was performed and then the image was thresholded again. After this step, pixel clusters smaller than the area of a single platelet were removed. The resulting images were used to calculate the surface coverage by dividing the number of pixels with values greater than zero by the total number of pixels. Finally, individual thrombi were identified, and the mean thrombus area was estimated. Figure 8c shows the results of the image analysis procedure: The central image represents the output of the thresholding operation, i.e., the segmented binary image from which surface coverage was calculated, while the image at the bottom shows the result of the object identification algorithm.

### 4.6. Statistical Analysis

Statistical analysis of the results was performed using GraphPad Prism 6 (GraphPad Software, Inc., CA, USA). Normal distribution of data was tested with the Shapiro–Wilk normality test. The One-way Analysis of Variance (ANOVA) test or t-tests were used when the normality hypothesis was satisfied. Conversely, non-parametric Kruskal–Wallis or Wilcoxon tests were performed. Statistical significance was assumed for *p*-values lower than 0.05.

## Abbreviations

MFC	microfluidic flow chambers
WSR	wall shear rates
ASA	acetylsalicylic acid
RT	room temperature
COX-1	cyclooxygenase-1
PDMS	polydimethylsiloxane
PBS	phosphate-buffered saline
K-EDTA	potassium ethylenediaminetetra-acetic acid
